# Case of Apoplexy

**Published:** 1807-05

**Authors:** Lyman Spalding

**Affiliations:** Portsmouth, New Hampshire


### Case *of* Apoplexy, *communicated by* Lyman Spalding, M. D. &c. *of Portsmouth, New Hampshire.*

In the month of November 1800, I visited Capt. John Yeaton, of this town, aged 22 years; he had a large swelling on the right lumbar region, extending forward into the hypochondria. This tumour was six inches in diameter, ter, rising two inches above the natural surface. By its elasticity, colour, and appearance, I thought it a lymphatic tumour.

I saw him but a few times before he went to sea, and by the month of November 1801, made two voyages to the West Indies, when he applied to me again. The tumour had now broke, and discharged a thin, viscid, glairy matter, from an orifice the size of a quill, situated immediately beneath the last false rib; into this orifice a probe might be introduced two or three inches in a direction upward and backward.

Innumerable applications were made to heal this ulcer, which was now become fistulous, but all in vain. As the spring opened, he began to recruit, by the aid of wine, bark, iron, diet drinks, &c. In May he found himself able to go to sea, enjoying tolerable health. In 1804, a similar tumour appeared on the left side, in precisely the same relative situation, and ended in a similar fistulous ulcer, from both of which there has been a constant drain.

In the summer of 1806, on account of bad health, he returned to this town, from the State of Virginia, where he had resided for several years, and from whence he had sailed in the West India trade.

The Physicians who saw him, supposed the liver was affected; they formed their prognosis from the torpor of the bowels; great costiveness, suffusion of bile, and his ejecting from the stomach, every morning, about half a pint of its contents mixed with bile; pulse small, respiration a little impaired when at ease, by exercise becoming laborious; often felt a degree of swimming in his head; at times stupid or comatose in a small degree; appetite bad, tongue furred a little, bowels swoln and painful to touch, skin dry, paucity of urine, high coloured and seldom passed, sleep natural. For these symptoms, blisters, calomel, and the warm bath were prescribed.

On the 5th of November 1806, at ten o'clock, A. M. he had a very strong convulsive fit, that lasted fifteen minutes, with stertorous breathing. The attending physician, Dr. Bracket, being out of town, Dr. J. H. Pierrepont and myself were called; we thought the case apoplectic, opened a vein, and drew eight ounces of blood. While we were in consultation a second fit appeared; another vein was opened, from which we drew eighteen ounces in a full stream; the pulse now rose, having been very small and depressed; gave a dose of calomel and jalap, and ordered an enema.

At

At the evening consultation, the attending physician present, the blood exhibited a very extraordinary appearance; a very thick buff, so much cupped as to appear like an inverted mushroom floating in serum, and occupied not more than one third part of the diameter of the bowl. All the blood that was drawn exhibited the same appearances, whether from a large orifice, in a full stream, or trickled off the arm. This evening he was untractable, and while the attendants were endeavouring to administer an enema, he had a third fit; bled a third time.

Nov. 6. Slept much, with stertorous breathing; bled, and took senna and manna.

7. A fit this morning; slept some, talks incoherently, breathing laborious, pulse rather freer; bled.

8. Slept comfortably, appeared better, conversed rationally, took some food, had several stools; blisters, musk, and ether.

9. Not so well as yesterday, bowels swollen, passed his stools and urine involuntarily, talks incoherently; blisters on the head; musk, ether, and laudanum.

10. Pulse threaded, slept some, passed his stools involuntarily, puked, talks incoherently.

11. Had two fits last night, one this morning, and died at five o'clock, breathing stertorously.

Is it not more than probable that the first symptoms, in this case, indicated a predisposition to apoplexy, and that the succeeding concatenated therewith, making in the whole, one chain of cause and effect?

How far the two fistulous ulcers on the back, paved the way to, or were connected with the apoplexy, I am not able to say: neither am I fully satisfied as to the nature and extent of those ulcers, nor of the parts concerned, never having seen a similar case. It is very much to be lamented that the friends would not suffer the body to be opened, to ascertain the parts diseased.

